# Improving Women’s Sexual and Reproductive Health in a Psychiatric Inpatient Setting Quality Improvement Project: Development and Implementation of a Women’s Physical Health Clinic in a Psychiatric Hospital in North London

**DOI:** 10.1192/bjo.2025.10371

**Published:** 2025-06-20

**Authors:** Paul Gyimah, Tomilola James, Saira Chowdhary, Chiara Petrosellini, Monika Gorny

**Affiliations:** 1 Highgate Mental Health Centre, North London NHS Foundation Trust, London, United Kingdom; 2 University College London, Division of Psychiatry, London, United Kingdom

## Abstract

**Aims:** Following a pre-clinical survey of psychiatric female inpatients, it was highlighted that they found it challenging to access obstetric, gynaecological and sexual health investigations and management. It was also found that mental healthcare professionals in the same psychiatric unit had limited knowledge and awareness of women’s physical health issues. The aim of this QI project were to develop and establish a monthly women’s physical health clinic (WPHC) on an inpatient psychiatric hospital site, offering assessment, investigation and treatment by obstetricians and gynaecologists.

**Methods:** We have established a monthly WPHC occurring, since January 2024, on every 3rd Thursday of the month 1–5 pm at a psychiatric hospital in North London. It was run voluntarily by two local obstetrics and gynaecology (OBGYN) specialist registrars with a special interest in mental health. Specialised clinical equipment was sourced through central procurement. We developed a detailed referral pathway. This involved creating a referral form which would be emailed to all female wards and later screened. Patients accepted into the clinic were booked for roughly 45-minute slots based on priority. The OBGYN involvement included specialist investigations, treatments and liaison with patients’ GPs. In order to raise awareness of the WPHC with psychiatric inpatient staff and patients, we designed posters and information leaflets, sent weekly email reminders to the clinical team about the clinic referral procedures and raising awareness through trust induction, academic teaching, and the Resident doctors’ WhatsApp group.

**Results:** Referrals increased from 8 before May 2024 to 28 after implementing targeted interventions totalling 36 overall. While numbers increase initially, fluctuations occurred in subsequent months due to leave, strikes and staff shortages. Patient qualitative feedback obtained via surveys included requests for more frequent clinics (unmet need was even greater than anticipated), positive experience of a smooth service and complaints related to clinic delays linked to multiple factors. Staff feedback included satisfaction with the simplicity of the referral form, swift replies. Virtual clinics were suggested as a way of improving the access further, especially for advice regarding acutely unwell patients.

**Conclusion:** Our QI project data has demonstrated the importance of providing women with physical health care in a female psychiatric inpatient setting. The large increase in referrals following introduction of the WPHC highlights the unmet medical need for female psychiatric inpatients accessing obstetric, gynaecological and sexual health services. Our next steps will include securing funding for more regular, biweekly clinics, as the unmet need identified is greater than expected.

