# Improving Women’s Sexual and Reproductive Health in a Psychiatric Inpatient Setting Quality Improvement Project: Development and Implementation of a Women’s Physical Health Clinic in a Psychiatric Hospital in North London – CORRIGENDUM

**DOI:** 10.1192/bjo.2025.10906

**Published:** 2025-10-27

**Authors:** Chiara Petrosellini, Pollyanna Cohen, Monika Gorny, Paul Gyimah, Tomilola James, Neil Sarkar, Saira Chowdhary

In the original publication of this abstract, two co-authors were missing from the author list. The correct author list is as follows:

Chiara Petrosellini, Pollyanna Cohen, Monika Gorny, Paul Gyimah, Tomilola James, Neil Sarkar and Saira Chowdhary.

The abstract has been updated to amend this, and this corrigendum published.
